# Electrophysiologic effects of the *I*_K__1_ inhibitor PA‐6 are modulated by extracellular potassium in isolated guinea pig hearts

**DOI:** 10.14814/phy2.13120

**Published:** 2017-01-13

**Authors:** Gregory S. Hoeker, Mark A. Skarsfeldt, Thomas Jespersen, Steven Poelzing

**Affiliations:** ^1^ Biomedical Engineering and MechanicsCenter for Heart and Regenerative MedicineVirginia TechVirginia Tech Carilion Research InstituteRoanokeVirginia; ^2^ Department of Biomedical SciencesFaculty of Health and Medical SciencesUniversity of CopenhagenCopenhagenDenmark

**Keywords:** Action potential, conduction velocity, inward rectifier current, pentamidine, potassium, repolarization

## Abstract

The pentamidine analog PA‐6 was developed as a specific inward rectifier potassium current (*I*_K__1_) antagonist, because established inhibitors either lack specificity or have side effects that prohibit their use in vivo. We previously demonstrated that BaCl_2_, an established *I*_K__1_ inhibitor, could prolong action potential duration (APD) and increase cardiac conduction velocity (CV). However, few studies have addressed whether targeted *I*_K__1_ inhibition similarly affects ventricular electrophysiology. The aim of this study was to determine the effects of PA‐6 on cardiac repolarization and conduction in Langendorff‐perfused guinea pig hearts. PA‐6 (200 nm) or vehicle was perfused into ex‐vivo guinea pig hearts for 60 min. Hearts were optically mapped with di‐4‐ANEPPS to quantify CV and APD at 90% repolarization (APD_90_). Ventricular APD_90_ was significantly prolonged in hearts treated with PA‐6 (115 ± 2% of baseline; *P* < 0.05), but not vehicle (105 ± 2% of baseline). PA‐6 slightly, but significantly, increased transverse CV by 7%. PA‐6 significantly prolonged APD_90_ during hypokalemia (2 mmol/L [K+]_o_), although to a lesser degree than observed at 4.56 mmol/L [K+]_o_. In contrast, the effect of PA‐6 on CV was more pronounced during hypokalemia, where transverse CV with PA‐6 (24 ± 2 cm/sec) was significantly faster than with vehicle (13 ± 3 cm/sec, *P* < 0.05). These results show that under normokalemic conditions, PA‐6 significantly prolonged APD_90_, whereas its effect on CV was modest. During hypokalemia, PA‐6 prolonged APD_90_ to a lesser degree, but profoundly increased CV. Thus, in intact guinea pig hearts, the electrophysiologic effects of the *I*_K__1_ inhibitor, PA‐6, are [K+]_o_‐dependent.

## Introduction

The inward rectifier potassium current (*I*_K1_) is an important regulator of the cardiac action potential, serving to stabilize the resting membrane potential (Sakmann and Trube 1984; Tourneur 1986), and contributing to late repolarization (Kass et al. 1990; Ibarra et al. 1991). The molecular basis of cardiac *I*_K1_ is attributed to the Kir2.x subfamily of inward rectifier potassium channel proteins (Dhamoon and Jalife 2005), which are strongly regulated by extracellular potassium concentration ([K^+^]_o_). For instance, hypokalemia is known to shift the reversal potential for *I*_K1_ to a more negative potential and reduce the slope conductance of the inward current (resulting in a decreased peak density of *I*_K1_), as well as hyperpolarize the resting membrane potential (Scamps and Carmeliet 1989; Shimoni et al. 1992; Hirota et al. 2000), which together alters sodium channel availability and cardiac excitability. Studies have suggested that *I*_K1_ plays a critical role in modulating cardiac excitability and the incidence of arrhythmias including congenital atrial fibrillation (Deo et al. 2013), catecholaminergic polymorphic ventricular tachycardia (Barajas‐Martinez et al. 2011), ventricular fibrillation (Warren et al. 2003), and arrhythmias associated with Andersen‐Tawil syndrome type I and short QT syndrome 3 (see Anumonwo and Lopatin (2010) for review). Furthermore, hypokalemia has been suggested to exacerbate conduction abnormalities, with reports of an increased risk of ventricular arrhythmias in Brugada patients (Araki et al. 2003; Notarstefano et al. 2005). Similarly, during hypokalemia patients with Andersen‐Tawil syndrome type 1 have more pronounced ECG changes (Zhang et al. 2005), a greater burden of premature ventricular contractions (Tawil et al. 1994; Nichols et al. 1996), and an increased occurrence of ventricular arrhythmias (Tawil et al. 1994; Tristani‐Firouzi et al. 2002). Lastly, in heart failure, which is associated with a loss of *I*_K1_ function (Kaab et al. 1996), both the complex pathologic state and common therapies can lead to electrolyte disturbances including hypokalemia (Leier et al. 1994). Thus, regulation of *I*_K1_ and potassium homeostasis has significant clinical implications for cardiac conduction and arrhythmogenesis.

Despite several decades of recognizing the importance of *I*_K1_ for cardiac function, the lack of specific and efficacious agonists/antagonists for Kir2.x channels has slowed progress toward understanding the physiologic and pathophysiologic roles of *I*_K1_ in the heart. Pharmacologic compounds targeting *I*_K1_ generally lack specificity for Kir2.x channels, or have toxic side effects that prohibit their use in vivo (de Boer et al. 2010; Bhoelan et al. 2014). Recently, seven analogs of the diamine antiprotozoal drug pentamidine were shown to inhibit *I*_K1_ at nanomolar concentrations. The sixth analog (PA‐6) was shown to have high efficiency and specificity for inhibition of the Kir2.x‐mediated current (i.e., *I*_K1_) (Takanari et al. 2013). In isolated cardiac myocytes, PA‐6 was previously shown to increase action potential duration (APD) (Takanari et al. 2013). Additionally, 200 nm PA‐6 prolonged APD in ventricular myocardium of isolated rat hearts (Skarsfeldt et al. 2016).

Previously, we demonstrated that partially inhibiting *I*_K1_ with BaCl_2_ prolongs ventricular APD and increases conduction velocity (CV) in ventricular myocardium of isolated guinea pig hearts (Poelzing and Veeraraghavan 2007; Veeraraghavan and Poelzing 2008), whose action potential morphology more closely mimics human action potentials than those of rats and smaller rodents. However, barium is known to have multiple off‐target effects, which could confound these findings (Lesage et al. 1995).

The aim of this study was to investigate the effects of the selective *I*_K1_ inhibitor, PA‐6, on action potential repolarization and conduction in an intact guinea pig heart preparation during normo‐ and hypokalemia. In this study, we demonstrate that in Langendorff‐perfused adult guinea pig hearts, inhibiting *I*_K1_ alone prolongs ventricular repolarization but does not substantially alter conduction. However, under hypokalemic conditions, which itself prolongs APD and decreases CV, treatment with PA‐6 resulted in further APD prolongation and increased CV.

## Materials and Methods

### Animals

Animal care and experimental procedures were approved by the Institutional Animal Care and Use Committee at Virginia Polytechnic Institute and State University and conducted in compliance with the European convention for the protection of vertebrate animals used for experimental and other scientific purposes (Council of Europe No 123, Strasbourg 1985).

Male retired breeder, albino Hartley guinea pigs (Hilltop Lab Animals, Scottdale, PA; *n* = 29, approximately 900–1200 g, 13–20 months old) were placed in an induction chamber and anesthetized with 5% isoflurane mixed with 100% oxygen at 3 L/min. After losing consciousness, the animal was removed from the induction chamber and masked with 3–5% isoflurane mixed with 100% oxygen at 4 L/min. Once in a surgical plane of anesthesia, a thoracotomy was performed, the heart was excised and rinsed in Tyrode's solution (see below for details on perfusate composition).

### Langendorff perfusion

After the heart was excised, the aorta was cannulated and perfused retrogradely with a modified Tyrode's solution containing (in mmol/L): NaCl 140, KCl 4.56, CaCl_2_ 1.25, dextrose 5.5, MgCl_2_ 0.7, and HEPES 10; pH was adjusted to 7.40–7.42 at 37°C using NaOH. The Tyrode's solution was bubbled with 100% oxygen and perfused at a constant flow to maintain a perfusion pressure of 40–55 mmHg. The atria were excised to reduce competitive stimulation, and the heart was placed in a custom‐made tissue bath where it was immersed in the perfusate and maintained at 37°C. Hearts were stimulated with a unipolar silver chloride wire positioned on the epicardium of the anterior left ventricle (LV) and paced at a basic cycle length (BCL) of 300 msec using a pulse width of 5 msec and current strength at 1.5× the diastolic threshold.

### Optical mapping

Cardiac motion was suppressed by adding the electromechanical uncoupler 2,3‐butanedione monoxime (BDM, 7.5 mmol/L) to the perfusate. The voltage sensitive dye di‐4 ANEPPS (7.5 *μ*mol/L; Biotium, Hayward, CA) was perfused into the heart for 10 min followed by a 10 min washout period before the start of the experimental protocol. Di‐4‐ANEPPS was excited by illuminating the anterior surface of the heart with a 150 W halogen light source (MHAB‐150 W, Moritex Corporation) and quartz fiber light guide (Moritex Corporation, Saitama, Japan) fitted with an excitation filter (center wavelength of 510/10 nm; Semrock, Rochester, NY). Fluoresced light was collected by a tandem lens assembly (0.63× magnification), transmitted through a 610 nm long pass filter (610FG01‐50(T257), Andover Corporation, Salem, NH), and detected by a 100 × 100 pixel CMOS camera (MiCAM Ultima‐L, SciMedia, Costa Mesa, CA) with a field of view of 15.9 × 15.9 mm^2^ (0.159 mm interpixel resolution). Fluorescence was recorded at a sampling rate of 1000 frames/sec.

### Reagents

Di‐4‐ANEPPS (5 mg) was dissolved in 1.3 mL of ethanol to create an 8 mmol/L stock solution. The pentamidine analog PA‐6 (C31H32N4O2) was synthesized by Syngene, Bangalore, India. PA‐6 (M.W. = 492.62) was dissolved in dimethyl sulfoxide (DMSO) and prepared as a 5 mmol/L stock solution. PA‐6 is known to interact with the cytoplasmic domain of Kir2.1 (Takanari et al. 2013), and data from rat hearts suggest that maximal, stable effects of PA‐6 occur between 45 and 90 min of exposure (Skarsfeldt et al. 2016). To maintain PA‐6 in solution and facilitate intracellular uptake over a sustained period, at the time of the experiment, 40 *μ*L of the PA‐6 stock solution was mixed with 40 *μ*L of Pluronic solution (1 g of Pluronic F‐127, Sigma‐Aldrich, dissolved in 5 mL of DMSO) and added to 1 L of Tyrode's solution for a final PA‐6 concentration of 200 nmol/L. For vehicle control (Veh) studies, 40 *μ*L of DMSO and 40 *μ*L of Pluronic solution were added to 1 L of Tyrode's solution.

### Experimental protocol

After the Di‐4‐ANEPPS washout period, steady‐state optical action potentials were recorded at baseline, and after 30 and 60 min of no treatment (time control, TC, *n* = 3) or treatment with either PA‐6 (200 nmol/L, *n* = 8) or Veh (DMSO + Pluronic, *n* = 5). In separate studies (PA‐6: *n* = 9, vehicle: *n* = 4), hearts were perfused with Tyrode's solution containing 2 mmol/L [K^+^] (vs. 4.56 mmol/L) and the experimental protocol was performed as described above.

### Data analysis

Fluorescence signals were binned 2 × 2, yielding an effective spatial resolution of 0.318 mm. Action potential activation times and 90% repolarization times were quantified as previously described (Girouard et al. 1996; Tian et al. 2004). APD at 90% repolarization (APD_90_) was calculated as the difference between the 90% repolarization and activation times. CV was quantified from contour maps of activation times transverse (CV_T_) and longitudinal (CV_L_) to fiber orientation as previously described (Entz et al. 2016). All data are presented as mean ± standard error of the mean. Two‐tailed, paired Student's t‐tests were used to compare mean APD_90_, CV_T_, and CV_L_ after treatment to corresponding baseline values within the same heart. Two‐tailed, unpaired Student's *t*‐tests were used to compare means between groups at a given time point. Differences were considered to be statistically significant for *P* < 0.05.

## Results

### Action potential prolongation with PA‐6 inhibition of *I*_K1_

**Figure 1:**
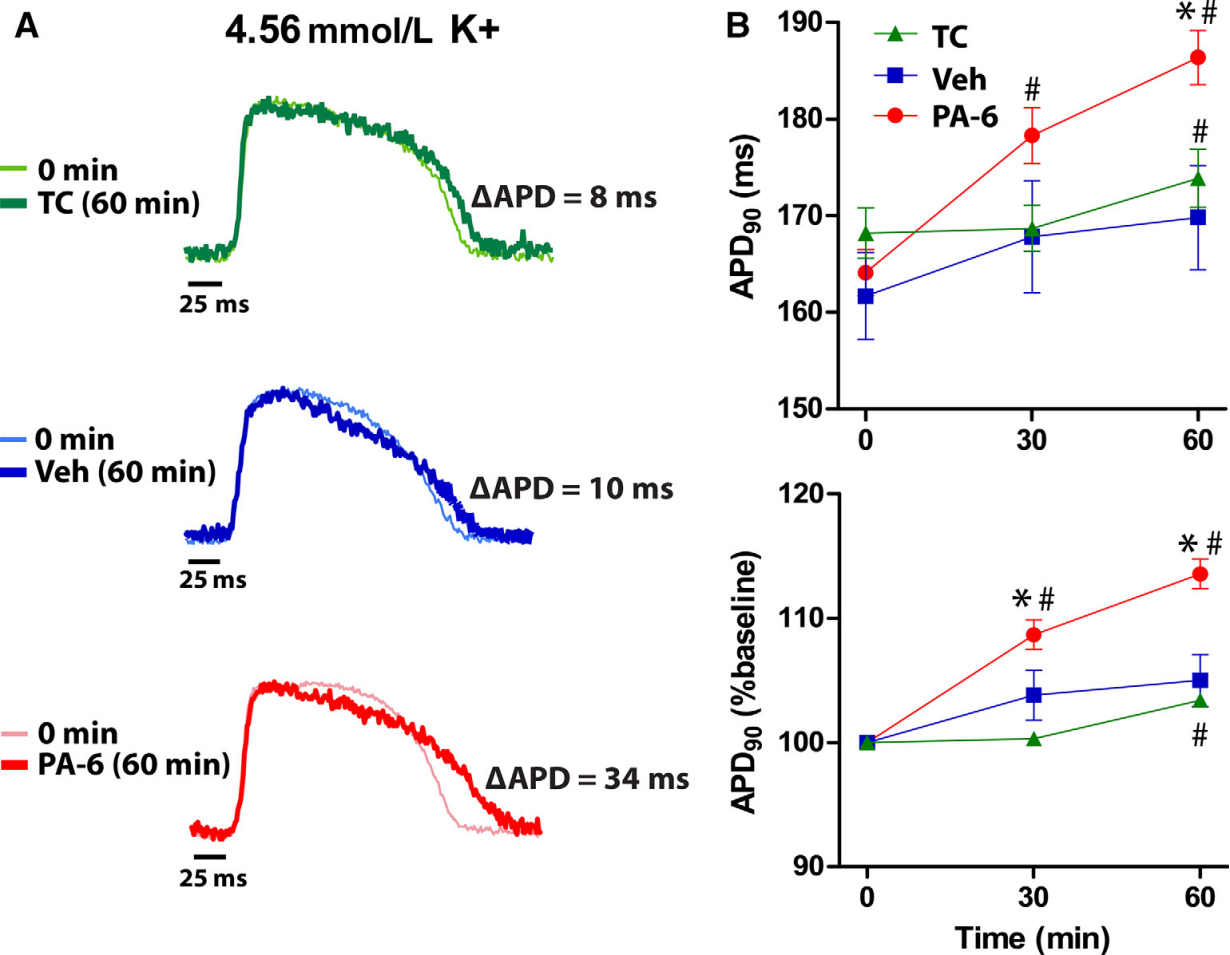
PA‐6 prolongs APD_90_ in normokalemic hearts. (A) Superimposed action potentials at 0 min (pretreatment) and after 60 min of time control (TC), or treatment with vehicle (Veh) or 200 nmol/L PA‐6 at [K^+^]_o_ = 4.56 mmol/L. The difference in APD_90_ values are shown as ∆APD in the inset. (B) Summary data of mean APD_90_ (top) and the change in APD_90_ (as a percent of baseline, bottom) over 60 min in each of the three treatment groups. *P < 0.05 versus Veh, ^#^ *P* < 0.05 versus baseline.

To investigate the effects of *I*_K1_ inhibition on ventricular repolarization, Langendorff‐perfused guinea pig hearts were treated with 200 nmol/L PA‐6 for 60 min and APD_90_ was measured from optical action potentials recorded at 0 (pretreatment), 30, and 60 min. The results from PA‐6 treated hearts (*n* = 8) were compared to time (TC, *n* = 3) and vehicle control hearts (Veh, *n* = 5) to assess the effects of preparation stability and the vehicle solvent (DMSO + Pluronic) on APD_90_. Shown in Figure 1A are superimposed action potentials from the same recording pixel at baseline (0 min) and 60 min for each treatment group: time control (TC), vehicle (Veh), and PA‐6. These traces demonstrate that over 60 min, the action potentials recorded in the time control and vehicle hearts were relatively unchanged. In contrast, there were marked changes in the action potential obtained after 60 min of treatment with PA‐6 (Fig. 1A, *bottom*). Specifically, PA‐6 appeared to affect late repolarization (phase 3 of the action potential), resulting in a prolonged action potential duration (∆APD_90_ = 34 msec). Over all experiments (*n* = 8), PA‐6 significantly prolonged APD_90_ (Fig. 1B) at both 30 (∆14.2 msec, 108.7% of baseline, *P* < 0.05) and 60 min (∆22.3 msec, 113.6% of baseline, *P* < 0.05) of treatment. Over the course of 60 min, a small (∆5.7 msec, 103.4% of baseline) but statistically significant APD_90_ prolongation was observed in untreated hearts (TC). At 60 min, APD_90_ in PA‐6 treated hearts was significantly longer than both Veh and TC hearts. There were no significant differences in APD_90_ between Veh and TC at any of the time points. These data demonstrate that PA‐6 increases APD_90_ independent of any effects due to time or exposure to vehicle.

### Negligible effect of PA‐6 on conduction velocity

**Figure 2:**
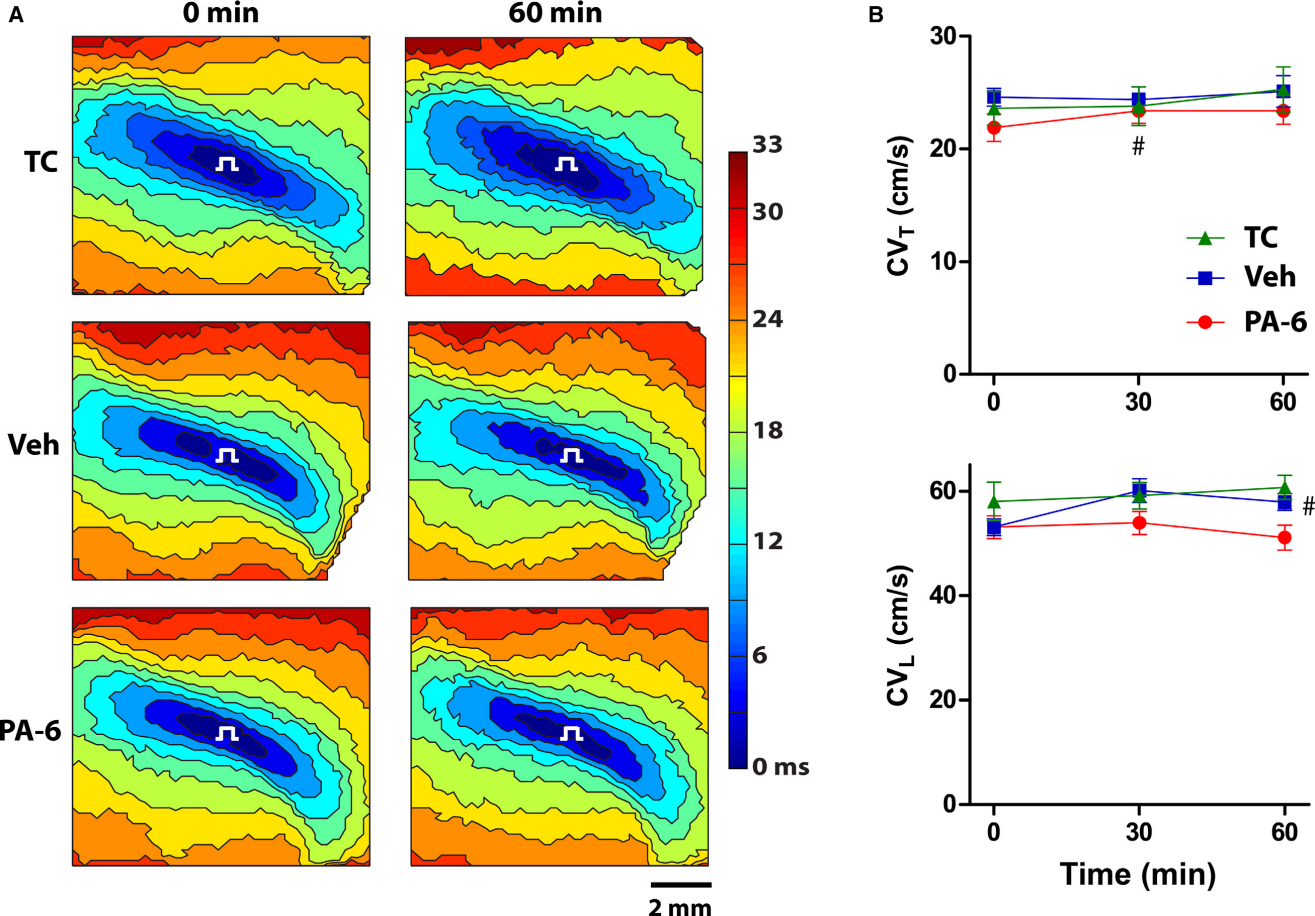
Effect of PA‐6 on CV in normokalemic hearts. (A) Representative contour maps of action potential activation times at 0 min (pretreatment) and 60 min of time control (TC), or treatment with vehicle (Veh) or 200 nmol/L PA‐6 at [K^+^]_o_ = 4.56 mmol/L. Each isochrone represents a 3 msec change in activation time. The pacing symbol at the center of each map indicates the site of stimulus delivery. (B) Summary data of mean CV_T_ (top) and CV_L_ (bottom) over 60 min in each of the three treatment groups. *P < 0.05 versus Veh, ^#^ *P* < 0.05 versus baseline.

We previously demonstrated that 10 *μ*mol/L BaCl_2_ can increase cardiac conduction (Veeraraghavan and Poelzing 2008). In order to assess the effect of a more potent and specific inhibitor of *I*_K1_ on transverse and longitudinal conduction, CV_T_ and CV_L_ were measured before and after treatment with 200 nmol/L PA‐6 during normokalemia ([K^+^] = 4.56 mmol/L). Representative maps of activation isochrones are presented in Figure 2A. These maps demonstrate that after 60 min, there was very little change in the activation patterns due to time (TC) or treatment with Veh or PA‐6. Summary data of CV (CV_T_ and CV_L_) revealed a very modest effect of inhibiting *I*_K1_ on conduction (Fig. 2B). Specifically, treatment with 200 nmol/L PA‐6 modestly, but significantly, increased CV_T_ by paired comparison from 21.9 ± 1.2 to 23.4 ± 1.1 cm/sec at 30 min (*P* < 0.05) and 23.4 ± 1.2 cm/sec at 60 min (*P* = 0.05). However, PA‐6 did not increase conduction relative to TC or Veh, as determined by unpaired statistical comparisons. Hence, neither CV_T_ nor CV_L_ were significantly different in hearts treated with PA‐6 compared to TC and Veh‐treated hearts at any time point.

### *I*_K1_ inhibition during hypokalemia

Lowering [K^+^]_o_ leads to a decrease in *I*_K1_ peak current density (Scamps and Carmeliet 1989), as well as changes in resting membrane potential and excitability (Shimoni et al. 1992). Previously, we demonstrated that hypokalemia differentially modulates ventricular myocardial electrophysiology during partial *I*_K1_ blockade with 10 *μ*mol/L BaCl_2_ (Poelzing and Veeraraghavan 2007). Therefore, we sought to test the effect of PA‐6 on repolarization and conduction during conditions of hypokalemia. Isolated hearts were equilibrated in Tyrode's solution containing 2 mmol/L [K^+^]_o_, and the experimental protocol was repeated as before with 200 nmol/L PA‐6 (*n* = 9) and Veh (*n* = 4). At baseline, hypokalemia by itself prolonged APD_90_ (202.4 ± 5.3 compared to 164.1 ± 1.9 msec at 4.56 mmol/L [K^+^]_o_, *P* < 0.05) and decreased CV_T_ (18.0 ± 1.8 compared to 23.1 ± 0.7 cm/sec at 4.56 mmol/L [K^+^]_o_, *P* < 0.05) and CV_L_ (37.0 ± 2.7 compared to 54.0 ± 1.4 cm/sec at 4.56 mmol/L [K^+^]_o_, *P* < 0.05).

**Figure 3:**
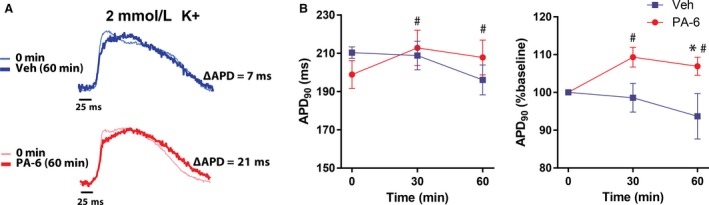
PA‐6 prolongs APD_90_ in hypokalemic hearts. (A) Superimposed action potentials at 0 min (pretreatment) and after 60 min of treatment with vehicle (Veh) or 200 nmol/L PA‐6 at [K^+^]_o_ = 2 mmol/L. The difference in APD_90_ values are shown as ∆APD in the inset. (B) Summary data of mean APD_90_ (left) and the change in APD_90_ (as a percent of baseline, right) over 60 min during treatment with PA‐6 or Veh. *P < 0.05 versus Veh, ^#^ *P* < 0.05 versus baseline.

Representative action potentials in Figure 3A demonstrate the action potential changes induced by PA‐6 during hypokalemia. While the action potential at baseline (0 min) is already longer than the corresponding action potentials in Figure 1A due to reduced [K^+^]_o_, subsequent treatment with 200 nmol/L PA‐6 resulted in further prolongation of APD_90_. In contrast, the action potentials from Veh‐treated hearts were nearly superimposable, with no discernable effect on repolarization. The time‐dependent effects of PA‐6 on APD_90_ during hypokalemia are summarized in Figure 3B. Within 30 min, PA‐6 significantly increased APD_90_ (∆13.9 msec, 109.3% of baseline, *P* < 0.05) and the prolongation persisted up to 60 min (∆8.9 msec, 106.9% of baseline, *P* < 0.05). This APD prolongation (as a percent change from baseline) was significantly less than that observed after 60 min of PA‐6 treatment with 4.56 mmol/L [K^+^]_o_ (6.9% vs. 13.6%, *P* < 0.05). The reduced effect of PA‐6 on APD prolongation during hypokalemia could possibly be due to partial inhibition of *I*_K1_ by the lowered [K^+^]_o_ prior to application of PA‐6. There were no significant differences in APD_90_ in Veh‐treated hearts at either 30 or 60 min.

**Figure 4:**
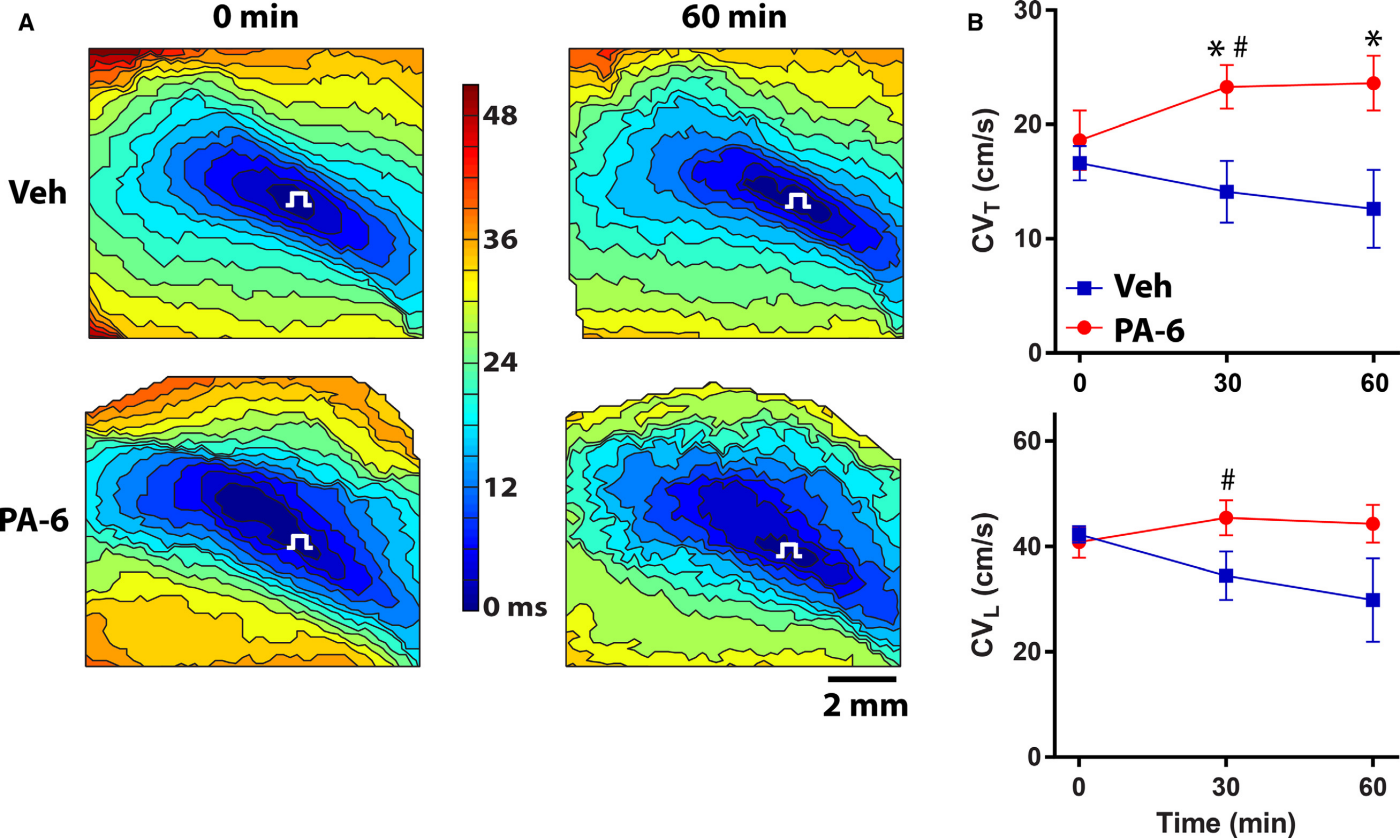
PA‐6 increases CV in hypokalemic hearts. (A) Representative contour maps of action potential activation times at 0 min (pretreatment) and 60 min of treatment with vehicle (Veh) or 200 nmol/L PA‐6 at [K^+^]_o_ = 2 mmol/L. Each isochrone represents a 3 msec change in activation time. The pacing symbol at the center of each map indicates the site of stimulus delivery. (B) Summary data of mean CV_T_ (top) and CV_L_ (bottom) over 60 min in each of the three treatment groups. *P < 0.05 versus Veh, ^#^ *P* < 0.05 versus baseline.

Comparing the representative baseline (0 min) activation maps at 2 mmol/L [K^+^]_o_ (Fig. 4A) to those at 4.56 mmol/L [K^+^]_o_ (Fig. 2A), it is apparent that there is crowding of isochrones during hypokalemia representing slower conduction due to the decrease in [K^+^]_o_. The pattern of activation and number of isochrones was relatively unchanged by 60 min of treatment with Veh. In contrast, the activation map after 60 min of PA‐6 revealed a marked effect on conduction, with far fewer isochrones (total activation time = 34 msec vs. 42 msec at baseline) and a corresponding increase in CV. In fact, the summary data presented in Figure 4B shows that for all experiments PA‐6 reversed the decrease in CV_T_ that was observed with hypokalemia, and restored CV_T_ to values observed under normakalemia (23.6 ± 2.4 cm/sec vs. 23.1 ± 0.7 cm/sec). Mean CV_T_ was greater with PA‐6 than Veh at both 30 min (23.3 ± 1.9 cm/sec vs. 14.1 ± 2.7 cm/sec, *P* < 0.05) and 60 min (23.6 ± 2.4 cm/sec vs. 12.6 ± 3.4 cm/sec, *P* < 0.05). A similar trend was observed for CV_L_, where mean CV_L_ after 30 minutes of treatment with PA‐6 was 45.4 ± 3.3 cm/sec (compared to Veh: 34.4 ± 4.6, *P* = 0.08) and after 60 min CV_L_ was 44.3 ± 3.6 cm/sec (compared to Veh: 29.8 ± 7.9, *P* = 0.09).

## Discussion

Recently, selective inhibition of *I*_K1_ with the pentamidine analog PA‐6 was shown to influence action potential repolarization and refractoriness in isolated rat hearts (Skarsfeldt et al. 2016). Prior to the development of PA‐6, our laboratory demonstrated that inhibiting *I*_K1_ with BaCl_2_ led to APD prolongation(Poelzing and Veeraraghavan 2007) and enhanced conduction(Veeraraghavan and Poelzing 2008) in Langendorff‐perfused guinea pig hearts. In this study, using the same experimental model, we found that 60 min of treatment with 200 nmol/L PA‐6: (1) significantly prolonged ventricular APD_90_; (2) had minimal to no effect on CV_T_ or CV_L_ during normokalemia; (3) prior to administration of PA‐6, hypokalemia (2 mmol/L [K^+^]_o_) significantly prolonged APD_90_ and decreased CV_T_ and CV_L_; (4) during hypokalemia, PA‐6 prolonged APD_90_ further; and (5) in contrast to normokalemia, treatment with PA‐6 during hypokalemia significantly increased CV_T_ and demonstrated a similar trend in CV_L_.

A role for *I*_K1_ in cardiac repolarization has been demonstrated by studies conducted in a number of species, using a variety of pharmacologic agents to inhibit Kir2.x [e.g. BaCl_2_ (Poelzing and Veeraraghavan 2007; Wu et al. 1999; Baiardi et al. 2003), MS‐551 (Nakaya et al. 1993; Sen et al. 1998) RP58866 (Rees and Curtis 1993), RP62719 (Williams et al. 1999; Biliczki et al. 2002), chloroquine (Noujaim et al. 2010), tamoxifen (He et al. 2003), carbon monoxide (Liang et al. 2014), and cesium (Morita et al. 2007)]. Related studies have consistently found that inhibiting *I*_K1_ prolongs the QT interval and APD, although the severity of the effect varies widely (Rees and Curtis 1993; Biliczki et al. 2002; He et al. 2003). Among these studies, those reporting the largest effect on APD predominantly utilized nonselective compounds or doses of inhibitors that are known to be nonselective for *I*_K1_. Thus, it is possible that nonspecific effects influenced these findings, and that they represent an overestimation of the role of *I*_K1_ in normal cardiac repolarization.

In Langendorff‐perfused guinea pig hearts, we found that 60 min of treatment with 200 nmol/L PA‐6, a dose which has been shown to inhibit *I*_K1_ by 77–100% while having no effect on *I*_Na_, *I*_Ca_, *I*_to_, *I*_Kr_, or *I*_Ks_ (Takanari et al. 2013), prolonged APD_90_ by 14%. Previously, 200 nmol/L PA‐6 was shown to increase APD_90_ by 74% in Langendorff‐perfused rat hearts (Skarsfeldt et al. 2016). However, it is difficult to directly compare APD prolongation from studies in rat hearts to those of humans and other larger mammals with more pronounced action potential plateaus, as the composition and time course of their respective repolarizing currents are clearly distinct from one another. As a result, APD_90_ in ventricular guinea pig myocardium is fourfold longer than in rat at baseline conditions [164 msec vs. 42 msec (Skarsfeldt et al. 2016)]. Interestingly, the absolute change in mean APD_90_ induced by PA‐6 was similar in the two species (22.3 msec in guinea pig, 30.8 msec in rat). However, since late repolarization (where *I*_K1_ is active) constitutes a greater percentage of total APD in rat than guinea pig, the resulting percent difference in APD prolongation for a given absolute change is much larger in rat myocardium. A review of the literature supports the assertion that APD prolongation due to *I*_K1_ inhibition is enhanced in species with no action potential plateau (e.g., rat and mouse) relative to species with an action potential plateau (e.g., guinea pig, rabbit, canine, primate, and human) (Rees and Curtis 1993; Williams et al. 1999; Baiardi et al. 2003; Noujaim et al. 2010; Nagy et al. 2013). Accordingly, in isolated canine adult‐ventricular cardiomyocytes, 200 nmol/L PA‐6 increased APD_90_ by 34% (Takanari et al. 2013), which is similar to our findings in guinea pig. Using arguably the most commonly used approach for inhibiting *I*_K1_ (i.e., 10 *μ*mol/L BaCl_2_), we previously observed a 15–25% prolongation of APD in isolated guinea pig hearts, similar to the effect we are currently reporting with PA‐6. Thus, in larger mammals with action potential morphologies similar to those in human, the effect of PA‐6 on cardiac repolarization appears to be less than observed in smaller rodent species which lack a prominent plateau.

Given that *I*_K1_ plays a critical role in determining the resting membrane potential, it has been postulated that *I*_K1_ could oppose the depolarizing current through voltage‐gated sodium channels (i.e. *I*_Na_) during the early phase of AP activation. Consequently, inhibiting *I*_K1_ would lead to an increase in cardiac CV. Alternatively, decreased *I*_K1_ could potentially raise the resting membrane potential, resulting in sodium channel inactivation, thereby leading to a decrease in CV. However, the effect on the ventricular resting membrane potential following *I*_K1_ blockade has in most studies has been found to be either minor or undetectable, suggesting a resting membrane potential reserve of other potassium currents (see van der Heyden and Jespersen (2016) for review). Despite a well‐recognized role for *I*_K1_ modulation of cardiac excitability, surprisingly few studies have directly tested the prevailing theories of the effect of *I*_K1_ inhibition on ventricular conduction. Escande et al. (1992) saw no change in CV with RP62719, while Noujaim et al. (2010) reported a 35% decrease in CV with chloroquine. However, both of these *I*_K1_ inhibitors have been demonstrated to block other potassium currents at the doses tested, as well as sodium and calcium currents in the case of chloroquine (Jurkiewicz et al. 1996; Yang et al. 1999; Fujita and Kurachi 2000). In support of the theory that *I*_K1_ opposes *I*_Na_ depolarization, we have previously reported that 10 *μ*mol/L BaCl_2_ increased CV_T_ by approximately 25% (+6 cm/sec) (Veeraraghavan and Poelzing 2008). The corresponding increase in CV_T_ in this study with 200 nmol/L PA‐6 was 7% (+1.5 cm/sec). This is consistent with a lack of an effect of 200 nmol/L PA‐6 on resting membrane potential in isolated canine cardiomyocytes (Takanari et al. 2013). Altogether, this suggests that less specific *I*_K1_ inhibitors, such as BaCl_2_, may alter CV due to off‐target effects, and/or on its own, selective inhibition of *I*_K1_ can significantly impact repolarization, but alone may not be sufficient to change cardiac excitability or appreciably alter CV.

To further compare our findings with PA‐6 to those with BaCl_2_, we repeated the experimental protocol under conditions of low [K^+^]_o_. Consistent with previous results (Poelzing and Veeraraghavan 2007), lowering [K^+^]_o_ to 2 mmol/L prolonged APD by approximately 25%. Treatment with 200 nmol/L PA‐6 prolonged APD by a further 4–7%. Importantly, hypokalemia alone significantly decreased CV_T_ by 22%, whereas subsequent treatment with PA‐6 increased CV_T_ by 27%, effectively reversing the conduction loss due to hypokalemia and restoring CV_T_ to normokalemic values (23.6 cm/sec vs. 23.1 cm/sec). To our knowledge, this is the first study to investigate the effects of *I*_K1_ inhibition on cardiac conduction under conditions of hypokalemia. Therefore, these data suggest that PA‐6 may actually rescue conduction slowing induced by hypokalemia. Lowering [K^+^]_o_ will hyperpolarize the resting membrane potential, leading to a delay in sodium channel activation and therefore a slowing of conduction. Subsequent *I*_K1_ blockade could potentially depolarize the membrane and restore normal resting membrane potential, thereby alleviating the conduction abnormality induced by hypokalemia. Which leads to the question—why does not PA‐6 not increase CV at normokalemia? Perhaps cardiomyocyte excitability is more sensitive to small shifts (a few mV) in membrane potential at more hyperpolarized potentials than at normal resting membrane potential. Alternatively, perhaps *I*_K1_ inhibition alone is insufficient to significantly affect resting membrane potential or CV, and a further perturbation, such as hypokalemia, is required before an effect is observed. Data from Takanari et al. (2013) would support the latter, given that no change in resting membrane potential was observed in isolated canine cardiomyocytes treated with 200 nmol/L PA‐6. Lastly, it is possible that PA‐6 inhibition of *I*_K1_ is [K^+^]‐dependent. It has been demonstrated that permeant ions (in this case K^+^) can influence ligand interactions with ion channels (Zhorov and Tikhonov 2013), and perhaps the greater effect of PA‐6 on CV at low [K^+^]_o_ could be explained by such an interaction. These provocative hypotheses warrant further testing, particularly as new small molecule *I*_K1_ inhibitors such as ML133 are being developed for use in vivo (Wang et al. 2011).

### Limitations

Due to the degradation of di‐4‐ANEPPS fluorescence signals over time, we limited our experimental protocol to 60 min of PA‐6 treatment. While PA‐6 has been demonstrated to have a stable effect on APD_90_ within 45 min of treatment in Langendorff‐perfused rat hearts, the ventricular effective refractory period increased up to 90 min (Skarsfeldt et al. 2016). Therefore, it is possible that longer exposure times could reveal more pronounced effects of *I*_K1_ inhibition than observed in this study. While the specificity of PA‐6 for *I*_K1_ has been rigorously tested in heterologous systems expressing cardiac ion channels from human and mouse, as well as in isolated canine cardiomyocytes (Takanari et al. 2013), specificity has not been tested in guinea pig cardiomyocytes.

## Conclusion

Under normokalemic conditions, the *I*_K1_ inhibitor PA‐6 significantly prolonged APD_90_ without substantially affecting CV. During hypokalemia, PA‐6 prolonged APD_90_, although to a lesser degree, and significantly increased CV. Thus, in isolated guinea pig hearts, the electrophysiologic effects of PA‐6 are [K^+^]_o_‐dependent. Furthermore, these results highlight the importance of using a selective inhibitor to investigate the role of *I*_K1_ in cardiac repolarization and conduction, as well as validating these results in a species with action potential morphologies similar to those in human. Given its superior selectivity for *I*_K1_ and advantageous safety profile, PA‐6 will serve as an important tool for advancing our understanding of the physiologic and pathophysiologic roles of *I*_K1_ in vivo.

## Conflict of Interest

None declared.
